# Seven-day dietary nitrate supplementation clinically significantly improves basal macrovascular function in postmenopausal women: a randomized, placebo-controlled, double-blind, crossover clinical trial

**DOI:** 10.3389/fnut.2024.1359671

**Published:** 2024-06-10

**Authors:** Jocelyn M. Delgado Spicuzza, Jigar Gosalia, Liezhou Zhong, Catherine Bondonno, Kristina S. Petersen, Mary Jane De Souza, Elmira Alipour, Daniel B. Kim-Shapiro, Yasina B. Somani, David N. Proctor

**Affiliations:** ^1^Integrative Vascular Physiology Lab, Integrative and Biomedical Physiology, Huck Institutes of Life Sciences, The Pennsylvania State University, University Park, PA, United States; ^2^Integrative Vascular Physiology Lab, Department of Kinesiology, College of Health and Human Development, The Pennsylvania State University, University Park, PA, United States; ^3^Nutrition and Health Innovation Research Institute, School of Medical and Health Sciences, Edith Cowan University, Joondalup, WA, Australia; ^4^Medical School, The University of Western Australia, Perth, WA, Australia; ^5^Cardiometabolic Nutrition Research Lab, Department of Nutritional Sciences, College of Health and Human Development, The Pennsylvania State University, University Park, PA, United States; ^6^Women’s Health and Exercise Lab, Department of Kinesiology, College of Health and Human Development, The Pennsylvania State University, University Park, PA, United States; ^7^Department of Physics, Wake Forest University, Winston-Salem, NC, United States; ^8^Faculty of Biological Sciences, Department of Biological Sciences, University of Leeds, Leeds, United Kingdom

**Keywords:** menopause, endothelium, nitric oxide, dietary nitrate supplementation, aging

## Abstract

**Introduction:**

Cardiovascular disease (CVD) is the leading cause of death in women, with increased risk following menopause. Dietary intake of beetroot juice and other plant-based nitrate-rich foods is a promising non-pharmacological strategy for increasing systemic nitric oxide and improving endothelial function in elderly populations. The purpose of this randomized, placebo-controlled, double-blind, crossover clinical trial was to determine the effects of short-term dietary nitrate (NO_3_^−^) supplementation, in the form of beetroot juice, on resting macrovascular endothelial function and endothelial resistance to whole-arm ischemia–reperfusion (IR) injury in postmenopausal women at two distinct stages of menopause.

**Methods:**

Early-postmenopausal [1–6 years following their final menstrual period (FMP), *n* = 12] and late-postmenopausal (6+ years FMP, *n* = 12) women consumed nitrate-rich (400 mg NO_3_^−^/70 mL) and nitrate-depleted beetroot juice (approximately 40 mg NO_3_^−^/70 mL, placebo) daily for 7 days. Brachial artery flow-mediated dilation (FMD) was measured pre-supplementation (Day 0), and approximately 24 h after the last beetroot juice (BR) dose (Day 8, post-7-day BR). Consequently, FMD was measured immediately post-IR injury and 15 min later (recovery).

**Results:**

Results of the linear mixed-effects model revealed a significantly greater increase in resting FMD with 7 days of BR_nitrate_ compared to BR_placebo_ (mean difference of 2.21, 95% CI [0.082, 4.34], *p* = 0.042); however, neither treatment blunted the decline in post-IR injury FMD in either postmenopausal group. Our results suggest that 7-day BR_nitrate_-mediated endothelial protection is lost within the 24-h period following the final dose of BR_nitrate_.

**Conclusion:**

Our findings demonstrate that nitrate-mediated postmenopausal endothelial protection is dependent on the timing of supplementation in relation to IR injury and chronobiological variations in dietary nitrate metabolism.

**Clinical trial registration:**

https://classic.clinicaltrials.gov/ct2/show/NCT03644472

## 1 Introduction

Cardiovascular disease (CVD) risk exponentially rises after menopause (1, 2), in part due to reductions in vascular function (3–5). Estrogen plays a pivotal role in maintaining homeostatic nitric oxide (NO) bioavailability in estrogen-replete premenopausal women (6, 7). Previous epidemiological data demonstrate that premenopausal women are protected from ischemic coronary artery disease compared with age-matched men; however, this protection is lost postmenopause (8). A major mechanism proposed to underlie menopause-induced vascular dysfunction is hypoestrogenemia and its NO-mediated vasodilator, vasoprotective, and antioxidant effects (9–12). The close association between CVD risk and estrogen deficiency highlights the clinical importance of cardiovascular health for postmenopausal longevity.

The presence of endogenous estrogen might not only affect basal endothelial function, but additionally, it can influence *endothelial cell resistance and resilience* following periods of tissue ischemia and subsequent reperfusion (i.e., ischemia–reperfusion (IR) injury) (13–17). Endothelial IR injury, which occurs during myocardial infarction, cardiac, and limb surgery, is defined by a temporary period of blood flow restriction and subsequent reperfusion, resulting in the production of damaging reactive oxygen and nitrogen species (RONS) (15). Premenopausal women in the late follicular phase of the menstrual cycle, when estradiol concentration is high, exhibit greater endothelial resistance to whole-arm IR injury compared to the early follicular phase when estradiol concentrations are low (14). The inverse association between serum estradiol and endothelial resistance is also supported by recent evidence from our laboratory, demonstrating that early-postmenopausal women, within 1–6 years of their final menstrual period (FMP), exhibit attenuated endothelial resistance to IR injury compared to premenopausal women despite comparable resting endothelial function (13). These findings support a role for the importance of endogenous estrogen-mediated endothelial protection.

It has long been hypothesized that estrogen therapy could be a primary prevention strategy to reduce CVD risk in postmenopausal women by mimicking the estrogen-replete premenopausal environment (18). However, evidence supports that the overall health risks of hormone therapy (HT, estrogen + progestin) exceed the vascular benefits in this population (19, 20). Additionally, the *timing hypothesis* emphasizes that hormone therapy has a more favorable effect in lowering future CVD events, in recently postmenopausal women compared to their more estrogen-deficient, chronologically older, late-postmenopausal (6+ years since the FMP) counterparts (18, 21, 22). The postmenopausal-stage variations in endothelial responsiveness to HT suggest that further investigation into non-pharmacological CVD targeted interventions for women in the later stage of menopause is necessary.

Nutraceutical interventions using nitrate-rich beetroot juice (BR) have emerged as a promising therapeutic strategy to increase systemic NO bioavailability and improve endothelial function in older, healthy, and high CVD-risk populations (23, 24). The entero-salivary pathway is a potential backup system to maintain homeostatic NO levels when the oxygen supply is limited. While endogenous NO production occurs via the L-arginine-NO synthase (NOS) pathway, the alternative exogenous dietary nitrate (NO_3_^−^)–nitrite (NO_2_^−^)–NO pathway relies on nitrate-rich foods such as arugula, spinach, and beetroot to enhance systemic NO bioavailability (25, 26). Within the entero-salivary circulation, nitrate-reducing bacteria in the oral cavity facilitate the reduction of nitrate to nitrite (26). Subsequently, the conversion of nitrite to NO is favored under ischemia-induced acidic conditions (i.e., low pO2 and pH) (24), such as occurs during IR injury. Therefore, nitrate-rich beetroot juice supplementation may represent an efficacious nutraceutical approach to increase NO bioavailability and subsequent endothelium-dependent vasodilation (23, 27).

Previously, we showed that early-postmenopausal endothelial resistance to IR injury significantly improved approximately 100 min after a single dose of nitrate-rich beetroot juice (BR_nitrate_). Whether macrovascular protection against IR injury is maintained 24 h following short-term BR_nitrate_ supplementation in postmenopausal women remains unexplored.

Accordingly, to extend our previous acute supplementation work, the primary aim of this investigation was to determine whether 7-day dietary nitrate supplementation confers postmenopausal stage-dependent variations in resting macrovascular function and endothelial resistance to whole-arm IR injury. In this double-blind, placebo-controlled, randomized crossover trial, we hypothesized that the effects of 7-day BR_nitrate_ supplementation would be maintained 24 h after the final dose (28) and would (1) increase resting endothelial function, (2) enhance endothelial resistance to IR injury, and (3) increase NO plasma metabolite concentrations to a greater extent in early-postmenopausal compared to late-postmenopausal women.

## 2 Materials and methods

### 2.1 Participants

Study participants were recruited from the Penn State campus and the greater surrounding State College, PA community, and provided written informed consent to participate in this registered clinical trial (NCT03644472). Out of the 54 women who were screened, 25 women completed all portions of the study. All procedures were approved by the Office of Research Protections at The Pennsylvania State University in agreement with the guidelines set forth by the Declaration of Helsinki.

Both early-postmenopausal [1–6 years following their final menstrual period (FMP)] and late- postmenopausal (>6 years following their FMP) women were recruited and staged based on the STRAW+1- criteria (29). Eligible participants did not have overt chronic disease as confirmed by a physician-reviewed medical history questionnaire and venous blood chemistry (hematological, liver, and kidney function). Eligible participants met the following criteria: resting brachial blood pressure < 130/80 mmHg, body mass index between 18.5 and 35 kg/m^2^, fasting plasma glucose <100 mg/dL or HbA1c <6.0%, fasting plasma low-density lipoprotein <160 mg/dL, non-smoker, not taking any cardiovascular medications or hormone therapy, and had not donated blood or blood products in the past 3 months. Following the determination of participant eligibility, volunteers were asked to complete four experimental study visits that consisted of vascular assessments and the IR injury protocol pre- and post-7-day nitrate-rich (BR_nitrate_) and nitrate-depleted (BR_placebo_) supplementation.

### 2.2 Overview of study design

Participants arrived at the Clinical Research Center (CRC) at The Pennsylvania State University between 7 am and 9 am having met the pre-testing requirements for all study visits: 12 h fasting from food and caffeine, 48 h without alcohol and dietary supplements, 24 h refraining from vigorous exercise, and 2 weeks without antioxidant supplements. Participants were asked to limit dietary nitrate intake throughout the 7-day supplementation period and were provided with a list of high-nitrate foods to avoid (leafy green vegetables, beetroot, watermelon, etc.). Participants were asked to record their diet 24 h prior to testing. After voiding and 10 min of seated rest, blood pressure and heart rate were measured in triplicate with a 1-min rest separating measurements. Participants were asked to refrain from using anti-bacterial mouthwash on experimental days and throughout supplementation as to preserve nitrate-reducing commensal bacterium in the oral cavity (30). Participants rested for an additional 10 min in a supine position after which brachial–ankle pulse-wave velocity was measured in triplicate with a 1-min rest between measurements (VP2000, Colin Medical). A baseline venous blood draw was taken from the left arm. Participants then walked to another room for vascular ultrasound assessments. After at least 10 min of supine rest, a baseline vascular assessment was conducted using brachial artery imaging with Doppler ultrasound to measure resting macrovascular function (see below for procedure details). Subsequently, participants consumed either NO_3_^−^-rich (BR_nitrate_, 300 mg NO_3_^−^ in 70 mL/6.4 mmol Beet-It Organic, James White Juice Company) or NO_3_^−^-depleted (BR_placebo_, 40 mg NO_3_^−^ per 70 mL/0.38 mmol nitrate-depleted Beet-It Organic, James White Juice Company) beetroot juice in random order, for 7 days. All vascular assessments were conducted on the same arm (right arm), in a dark, quiet, temperature-controlled (21°C) room, while following current guidelines (31). Eligible participants were randomly assigned in a 1-to-1 ratio to one of two randomization sequences by CRC nurse staff. The randomization sequence was generated electronically^1^ and stratified by the postmenopausal stage. The randomization schedule consisted of one block of 14 sequences for each postmenopausal stage. Blinding was achieved using identical-tasting interventions, and participants, as well as investigators, were kept unaware of the treatment sequences. The crossover design consisted of two treatment periods with a washout period of at least 2 weeks between them to minimize carryover effects. To best monitor adherence to the 7-day supplementation protocol, participants were asked to bring all the empty BR bottles to the post-supplementation visit.

**Figure 1:**
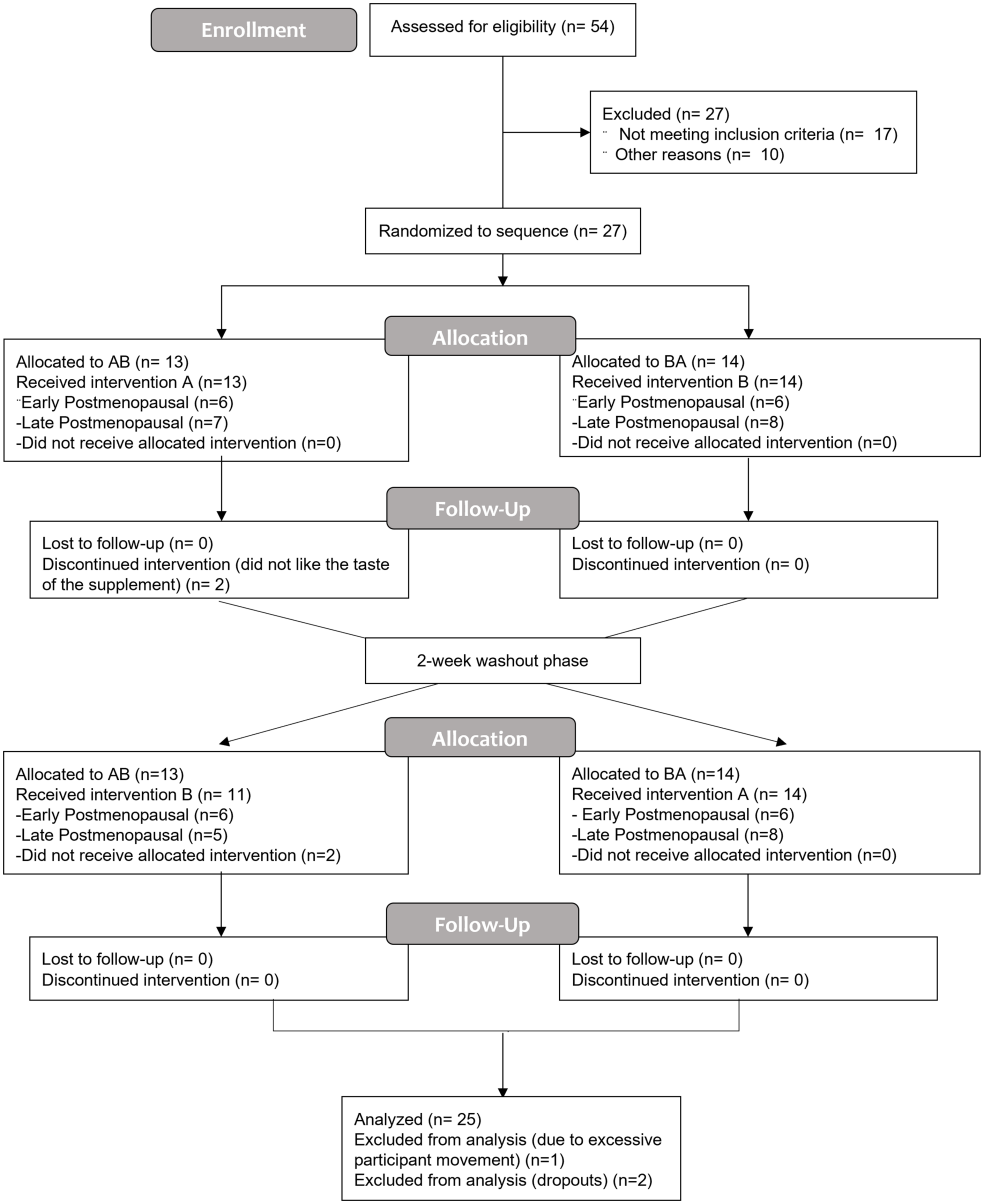
The CONSORT flow diagram is a summary of the randomized, double-blind, placebo-controlled, crossover design study to assess the influence of 7-day nitrate-rich and nitrate-depleted (placebo) beetroot juice supplementation on endothelial function at rest and after ischemia–reperfusion injury. Treatment period one (7 days); washout (2 weeks or more); and treatment period two (7 days). **(A)**, nitrate-depleted beetroot juice and **(B)**, nitrate-rich beetroot juice.

**Figure 2:**
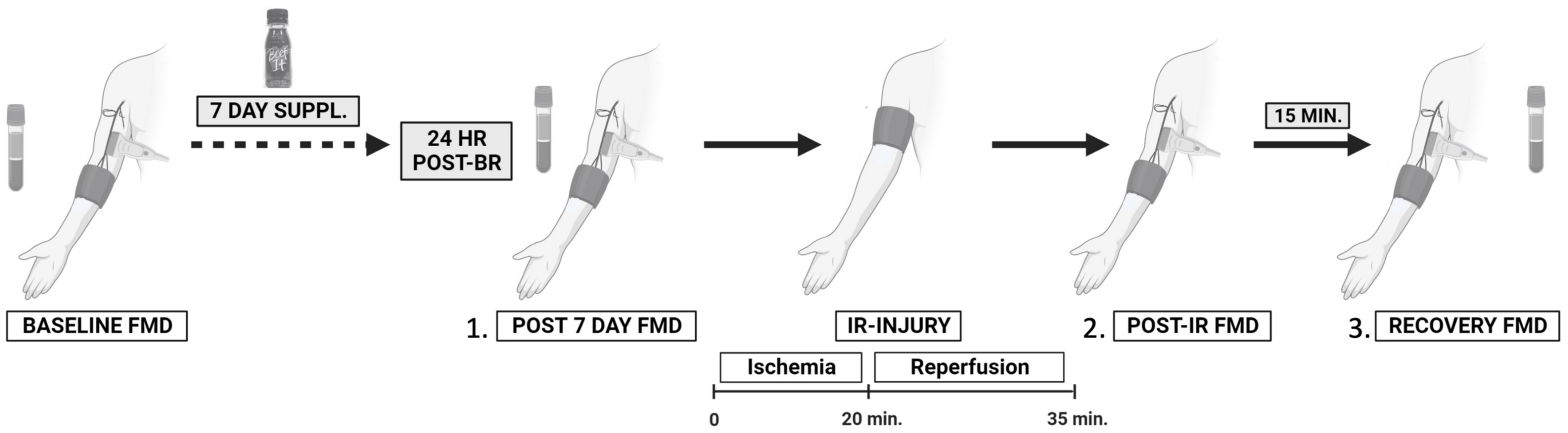
Schematic of the experimental protocol to assess the effects of 7-day beetroot juice supplementation on (1) resting endothelial function; (2) endothelial resistance against ischemia–reperfusion (IR) injury; and (3) recovery (resilience) from IR injury. Treatment period one (7 days); washout (2 weeks or more); and treatment period two (7 days). Created with BioRender.com.

Twenty four hours after consuming the last bottle of juice (Day 8), participants returned to the CRC. Resting seated blood pressure (left arm) and heart rate were measured followed by brachial–ankle pulse wave velocity. A venous blood sample was taken approximately 24 h following 7-day juice consumption. After at least 10 min of supine rest, a vascular assessment was conducted (see below for detailed procedures). Immediately following the vascular assessment, a rapid pneumatic cuff (Hokanson) was placed around the upper, right arm (as close to the axilla as possible) and inflated to 250 mmHg for 20 min followed by 15 min of reperfusion to induce temporary endothelial IR injury (15). The vascular assessments were repeated immediately after the reperfusion portion of the IR injury protocol (post-IR timepoint) and again 15 min later (30 min post-IR injury, recovery timepoint) to assess endothelial resistance and resilience, respectively (13). The final blood sample was taken at the end of all experimental visits (approximately 27–28 h after the last BR dose). A minimum 14-day washout period separated each week of supplementation. An overview of the study flow CONSORT diagram (Figure 1) and a schematic of the experimental protocol (Figure 2) are provided.

### 2.3 Plasma nitrate and nitrite analysis

Venous blood samples were collected into sodium heparin tubes (6-mL sodium heparin tubes, BD Vacutainer, Franklin Lakes, NJ, United States) and immediately centrifuged at 3,000 rcf (3,000 g) and 4°C for 4 min. Plasma was aliquoted and stored in a − 80°C freezer for later analysis. The ENO-20 analyzer was used to measure plasma NO_3_^−^ and NO_2_^−^ concentrations (sensitivity of 0.1 pmol for NO_3_^−^ and NO_2_^−^) according to the manufacturer’s protocol.

### 2.4 Dietary nitrate intake

Dietary nitrate intake was estimated from 24-h dietary recalls. Participants completed a total of four 24-h dietary recalls, which included two recall days (Day 0, i.e., 24 h prior to the baseline visit, and Day 7, i.e., 24 h prior to post-7-day supplementation visit) from each 7-day supplementation period for each treatment condition (two diet recalls from BR_placebo_ and two diet recalls from BR_nitrate_). Briefly, a nitrate food composition database for plant- (32) and animal-based (33) foods that also includes government analyses as part of national monitoring programs was used to quantify daily dietary nitrate consumption (mg/d). The estimated quantity of the plant- and animal-based foods consumed (g/d) was multiplied by the median nitrate value (mg/g) of each food. A 50% reduction in the assigned nitrate value was applied to the cooked plant-based foods to account for the effect of cooking (32); however, due to the limited number of eligible studies that detailed processing methods at the time of the database creation, the impacts of processing/cooking on nitrate content from animal-based foods were not included in the database (33). The total plant- and animal-based nitrate consumed per day was calculated by summing the nitrate values of each individual derived food (mg/d). To mitigate the potential influence of baseline dietary nitrate (mg/day 0) on post-supplementation FMD (day 8), baseline dietary nitrate intake was used as a covariate. Nitrate content from BR_nitrate_ (400 mg/d x 7 days) supplementation was not included in the analysis; only dietary nitrate (mg/d) from the background diet was included.

### 2.5 Ankle–brachial pulse wave velocity

After 10 min of supine rest, brachial–ankle pulse wave velocity (PWV) was measured in triplicate separated by 1 min using the VP2000 (Colin Medical) (34). According to the manufacturer’s instructions, blood pressure cuffs were placed securely around the participant’s upper arms and ankles, ECG electrodes were placed on the inner right and left wrists, and the phonocardiogram sensor on the proper rib-cage location. The automatic measurement was initiated and lasted approximately 45 s to 1 min.

### 2.6 Macrovascular function

#### 2.6.1 Experimental protocol

The endothelial macrovascular function was assessed by brachial artery flow-mediated dilation. Following 10 min of supine rest, the participant’s right arm was extended 80–90 degrees from their torso. Subsequently, a rapid inflation/deflation pneumatic cuff was placed around the forearm distal to the olecranon process. A multifrequency linear array probe attached to a high-resolution ultrasound machine (Phillips IU22) was used to capture longitudinal B-mode images of the brachial artery in the distal portion of the upper arm. Simultaneously, Doppler velocity was recorded at an insonation angle of 60 degrees and the sample volume was adjusted to the vessel size. Resting brachial artery diameter and blood velocity were recorded for at least 60–120 s. The pneumatic cuff was inflated to 250 mmHg for 5 min, and arterial lumen diameter and blood velocity were continuously measured during the occlusion period. Once the cuff was rapidly deflated, imaging continued for 3 min. Placement of the ultrasound transducer was marked on the participant’s arm to minimize differences in baseline arterial diameter between measurements. The same sonographer conducted all FMD tests and has a coefficient of variation (CV) of 16.9% for relative FMD and 1% for brachial artery baseline diameter. These values are in line with recommended expert values (31).

#### 2.6.2 Data analysis

The analysis of FMD data was conducted using live commercial edge-detection software (FMD Studio, Cardiovascular Suite 4, Quipu, Pisa, Italy) to analyze artery diameter, blood velocity, and shear rate. The optimal region of interest was analyzed by the same sonographer and was chosen based on image quality and clear distinction between the artery walls and lumen. FMD was calculated as the percent increase from baseline to peak diameter during reactive hyperemia using the following equation: $\frac{Peakdiameter-baselinediameter}{baselinediameter}\times 100$ on FMD Studio. To account for baseline diameter differences between postmenopausal groups, allometrically scaled FMD (Adjusted, Adj. FMD) was calculated as ln(peakdiameter)−ln(baselinediameter) (35, 36).

##### 2.6.2.1 Arterial hemodynamics

Shear rate (s^−1^, SR), defined as the frictional force exerted by blood flow, was calculated using the following formula: $\frac{meanbloodvelocity\;\left(\frac{cm}{s}\right)}{arterialdiameter\;\left(mm\right)}\times 4$ on FMD Studio (Cardiovascular Suite 4, Quipu, Pisa, Italy). Oscillatory shear index (OSI, a.u.) was calculated as baseline (during the 60s baseline FMD protocol) using the following equation: $\frac{negative\;SR\;\left(\sec^{-1}\right)}{\left[negative\;SR\;\left(\sec^{-1}\right)+positive\;SR\;\left(\sec^{-1}\right)\right]}.$ The shear rate area under the curve (SR AUC), defined as the area from the start of cuff inflation to the time of peak diameter, was calculated using FMD Studio (37). The blood flow is reported as a 30-s average proceeding cuff inflation and calculated by multiplying the cross-sectional area (πr^2^) of the artery with resting blood velocity. Peak blood flow, a surrogate measure of macrovascular reactive hyperemia, is reported as the highest 3-s average post-cuff release. RH blood flow AUC and velocity AUC, indirect measures of microvascular function, were calculated within the first minute following cuff release using the trapezoid method (Microsoft Corporation, Microsoft Excel Version 2,108).

##### 2.6.2.2 Ischemia–reperfusion injury protocol

Whole-arm endothelial IR injury was induced by placing a pneumatic cuff around the upper portion of the arm (as close to the axilla as possible) to sufficiently occlude the brachial artery. The cuff was inflated to 250 mmHg for 20 min. Post-cuff deflation, reperfusion occurred for 15 min and FMD was repeated immediately following reperfusion. The upper arm IR injury model is a non-invasive, well-established procedure employed in our laboratory and others to study human endothelial-mediated vascular IR injury (13, 15).

### 2.7 Statistical analyses

SPSS software (IBM Corp., version 28) was used to examine all data elements and perform statistical analyses. Non-normal data were log-transformed and considered normally distributed if the Shapiro–Wilk test statistic was not significant (*p* > 0.05). To investigate the effect of 7-day BR supplementation on resting FMD and Adj. FMD, and the absolute change in FMD from baseline to endpoint, the mixed model procedure (linear mixed-effects model, LMM) was used. Treatment, menopause stage, and the treatment by menopause stage interaction were modeled as fixed effects, the participant was included as a repeated factor, and the baseline outcome value and baseline dietary nitrate intake (24 h intake prior to the initiation of supplementation, Day 0) were included as covariates. To investigate the effect of 7-day BR supplementation on post-IR and recovery time points, the LMM was used with adjustment for baseline and habitual dietary nitrate intake (24-h intake prior to the initiation of supplementation, Day 0). Plasma [nitrate and nitrite] and brachial–ankle pulse wave velocity were evaluated using the mixed model procedure with adjustment for baseline plasma [nitrate and nitrite] and baseline brachial–ankle pulse wave velocity, respectively. Covariance structure selection was based on optimizing the fit statistics based on the Bayesian Information Criterion. Statistical significance was set at *p* < 0.05. For primary and secondary analyses, between-treatment differences at the post-7-day, post-IR, and recovery timepoints were assessed by the presence of main effects for treatment and a menopausal stage by treatment interaction. When a main effect for treatment or menopausal stage by treatment existed, the conservative Bonferroni correction method was used to adjust for multiple comparisons for all outcomes (i.e., unadjusted FMD, vascular hemodynamics, and resting blood pressure); however, as recommended per Atkinson and colleagues (35, 36), the Fisher’s least significant difference correction method was used to adjust for multiple comparisons for allometrically scaled FMD (Adj. FMD) data only. To account for missing blood samples and dietary recalls completely at random, the missing value analysis (MVA) procedure was conducted on the imbalanced plasma nitrate, nitrite, and dietary nitrate intake data sets (missing values for all data sets were < 5%) to confirm MVA *p* < 0.05 prior to imputing missing values using the series mean. Data are presented as least-squared means and standard deviations (SD), unless otherwise specified. Based on a previous study in our laboratory, 10 subjects provided 80% power, and an effect size of 0.5, to detect meaningful physiological and clinically relevant differences in FMD (≥ 1% increase, (38) following short-term dietary nitrate supplementation).

## 3 Results

### 3.1 Participants

Of the 27 participants that were randomized, 13 women (Early, *n* = 6; Late, *n* = 7) received the placebo intervention first and 14 women (Early, *n* = 6; Late, *n* = 8) received the nitrate-rich intervention first. Two participants did not continue with the study protocol due to not liking the taste of the supplement; their data were not included in the analysis. Following washout (2 weeks) and crossover, 11 women (Early, *n* = 6; Late, *n* = 5) received the nitrate-rich intervention first and 14 women (Early, *n* = 6; Late, *n* = 8) received the placebo intervention second. A total of 25 participants completed the entirety of the study protocol. One participant was removed from data analysis due to excessive participant movement and/or poor video quality during the FMD measures (Figure 1). The baseline characteristics for 24 postmenopausal women are presented by randomization sequence in Table 1. Results of the one-way ANOVA confirmed significant menopausal-stage (group) differences in years since menopause (Early Postmenopausal: 4 ± 2 years; Late Postmenopausal: 14 ± 5 years, *p* < 0.001) and chronological age (Early Postmenopausal: 56 ± 4 years; Late Postmenopausal: 63 ± 4 years, *p* < 0.001), providing supporting evidence that women were appropriately categorized into early- and late-postmenopausal stages.

**Table 1:** Baseline participant characteristics from the initial screening visit are presented as mea*n* ± SD by randomization sequence for 12 early- and 12 late-postmenopausal women.

	**Early-postmenopausal**		**Late-postmenopausal**	
**Characteristics**	**Placebo-nitrate-rich**	**Nitrate-rich-Placebo**	**Placebo-nitrate-rich**	**Nitrate-rich-Placebo**
*n*	6	5	6	8
Age (y)	56 ± 3	55 ± 5	64 ± 4	63 ± 5
Years since menopause	3 ± 2	4 ± 1	15 ± 5	13 ± 5
Body mass (Kg)	66.2 ± 10.2	68.0 ± 11.1	61.9 ± 8.4	60.0 ± 4.7
Height (cm)	166.3 ± 5.7	167.3 ± 6.2	162.0 ± 5.2	161.2 ± 3.9
BMI (Kg/m^2^)	24 ± 3	25 ± 4	26 ± 7	23 ± 1
Resting systolic BP (mmHg)	114 ± 11	110 ± 10	118 ± 8	116 ± 14
Resting diastolic BP (mmHg)	69 ± 12	64 ± 6	64 ± 6	68 ± 8
Resting HR (beats/min)	62 ± 7	63 ± 5	61 ± 7	64 ± 8
Total cholesterol (mg/dL)	214 ± 14	198 ± 46	207 ± 30	225 ± 20
LDL (mg/dL)	116 ± 18	125 ± 41	111 ± 30	127 ± 22
HDL (mg/dL)	73 ± 14	54 ± 6	79 ± 16	80 ± 20
Triglycerides (mg/dL)	79 ± 31	90 ± 28	80 ± 27	83 ± 44
Fasting glucose (mg/dL)	89 ± 7	90 ± 4	92 ± 9	94 ± 5
Hematocrit (%)	42 ± 1	40 ± 4	41 ± 3	40 ± 2
Hemoglobin (g/dL)	14 ± 0	13 ± 2	13 ± 1	13 ± 1
Physical activity (MET-week)	2,575 ± 2,994	3,261 ± 4,530	3,257 ± 2,582	2,567 ± 1,967
Parturition number	2 ± 1	3 ± 1	2 ± 1	2 ± 2
PWV (cm/s)	1,245 ± 95	1,209 ± 150	1,434 ± 270	1,469 ± 145

### 3.2 Arterial stiffness

Results of the LMM revealed no significant interaction or main effects for brachial–ankle PWV pre- and post-7-day supplementation, suggesting that treatment did not affect brachial–ankle PWV in either postmenopausal group.

### 3.3 Effects of BR_placebo_ and BR_nitrate_ on resting seated blood pressure and heart rate

No significant interaction or main effects of group or treatment were observed for resting seated systolic (SBP), diastolic (DBP) blood pressure, or heart rate (HR).

### 3.4 Dietary nitrate intake

There were no significant main or group*treatment interaction (*p* = 0.21) effects for dietary nitrate intake, suggesting that menopausal stage [Early PM: 73.55 ± 0.85 mg NO_3_^−^, CI 95% (51.37,105.21), Late PM: 103 ± 0.85 mg NO_3_^−^, 95% CI (72.68, 148.71), *p* = 0.17] and treatment [BR_placebo_: 86.83 ± 0.72 mg NO_3_^−^, CI 95% (64.52, 116.75), BR_nitrate_: 88.06 ± 0.72 mg NO_3_^−^, CI 95% (65.43,118.39), *p* = 0.93] did not affect dietary nitrate intake. Stratification of dietary nitrate by plant- and animal-based foods resulted in an average intake of 43.98 ± 95.59 mg NO_3_^−^/day (9.03 ± 14.56% contribution) and 9.02 ± 12.18 mg NO_3_^−^/day (1.87 ± 1.9% contribution), respectively.

### 3.5 Plasma nitrate and nitrite

**Figure 3:**
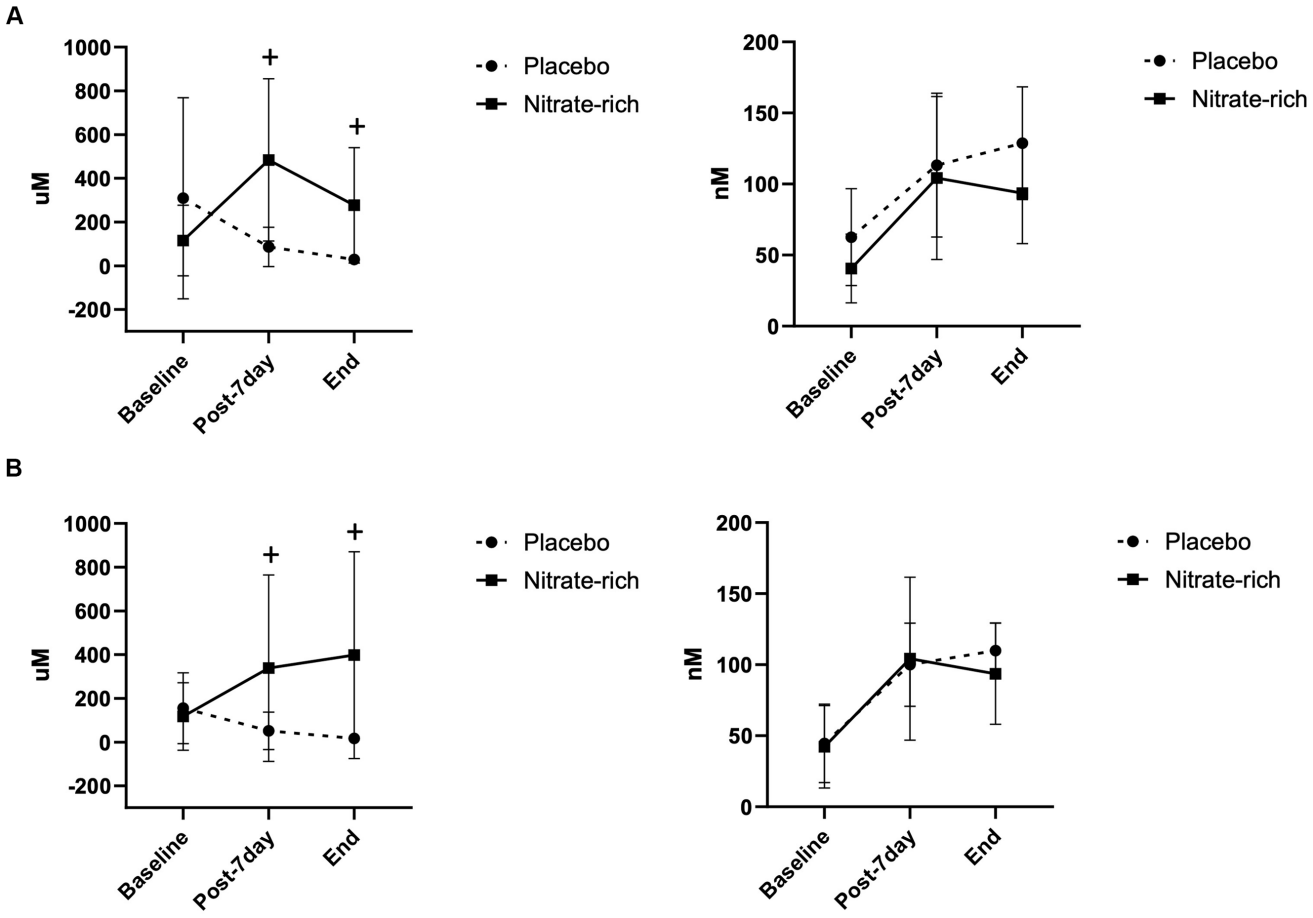
Plasma nitrate (left) and nitrite (right) concentrations at baseline (Day 0), 24 h following the last dose of BR (post-7-day, Day 8), and at the end of the experimental visits for BR_nitrate_ and BR_placebo_ in **(A)** 12 early-postmenopausal and **(B)** 12 late-postmenopausal women. Results of the LMM are represented as mea*n* ± SD. ^+^*p* < 0.05, from nitrate-depleted (placebo) beetroot juice.

Results of the LMM revealed a significant main effect of treatment (*p* < 0.001) for plasma [nitrate] (Figure 3); however, no significant group*treatment interaction was present. Pairwise comparisons showed significantly higher plasma nitrate concentration after 7-day supplementation with BR_nitrate_ and resulted in a mean difference of 342.4 ± 85.7 μM [CI 95% (169.5, 515.3), *p* < 0.001] between treatment conditions at the post-7-day timepoint. Plasma [nitrate] was significantly higher at the end timepoint with BR_nitrate_ compared to BR_placebo_. Pairwise comparisons resulted in a mean difference of 314.6 ± 80.7 μM [CI 95% (146.8, 482.4), *p* < 0.001] between treatment conditions at the end timepoint. No significant interaction or main effects were found for plasma nitrite concentration data across timepoints (Figure 3). In Figure 3, data are presented as mea*n* ± SD by menopausal stage; however, no significant main effect of group or group*treatment interaction was present.

### 3.6 Effects of BR_placebo_ and BR_nitrate_ on resting macrovascular function and endothelial resistance

**Figure 4:**
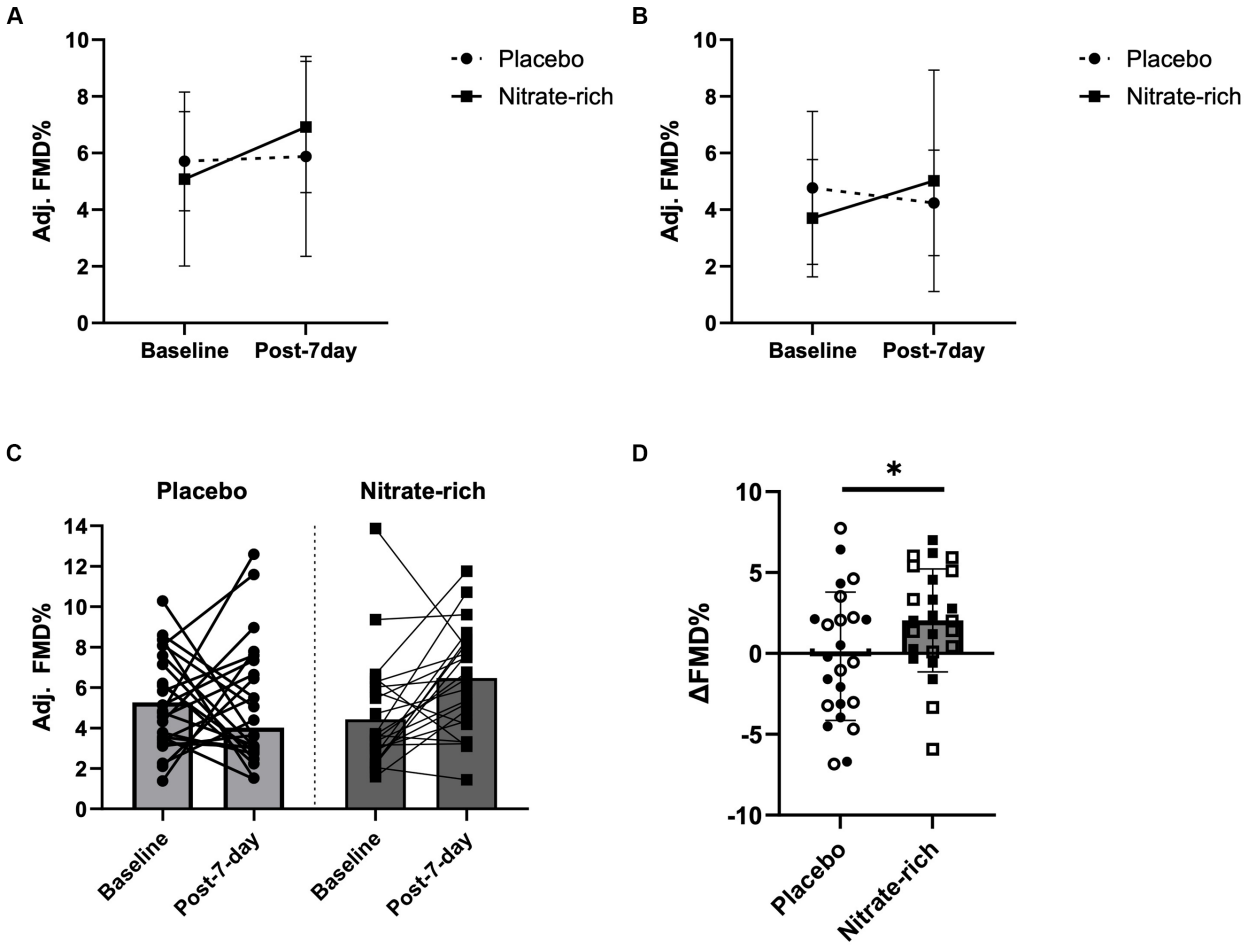
Brachial artery flow-mediated dilation at baseline and 24 h following the last dose of BR (post-7-day) for BR_nitrate_ and BR_placebo_ in **(A)** 12 early-postmenopausal and **(B)** 12 late-postmenopausal women. Results of the LMM are represented as mea*n* ± SD. Individual data **(C)** not grouped by postmenopausal stage (closed circles, placebo; closed squares, nitrate-rich) and **(D)** the absolute delta between baseline FMD and post-7-day FMD for both treatment conditions (open circles, early-postmenopausal BR_placebo_; open squares, early-postmenopausal BR_nitrate_, closed circles, late-postmenopausal BR_placebo_; closed squares, late-postmenopausal BR_nitrate_), ^*^p < 0.05, from nitrate-depleted (BR_placebo_) beetroot juice.

**Figure 5:**
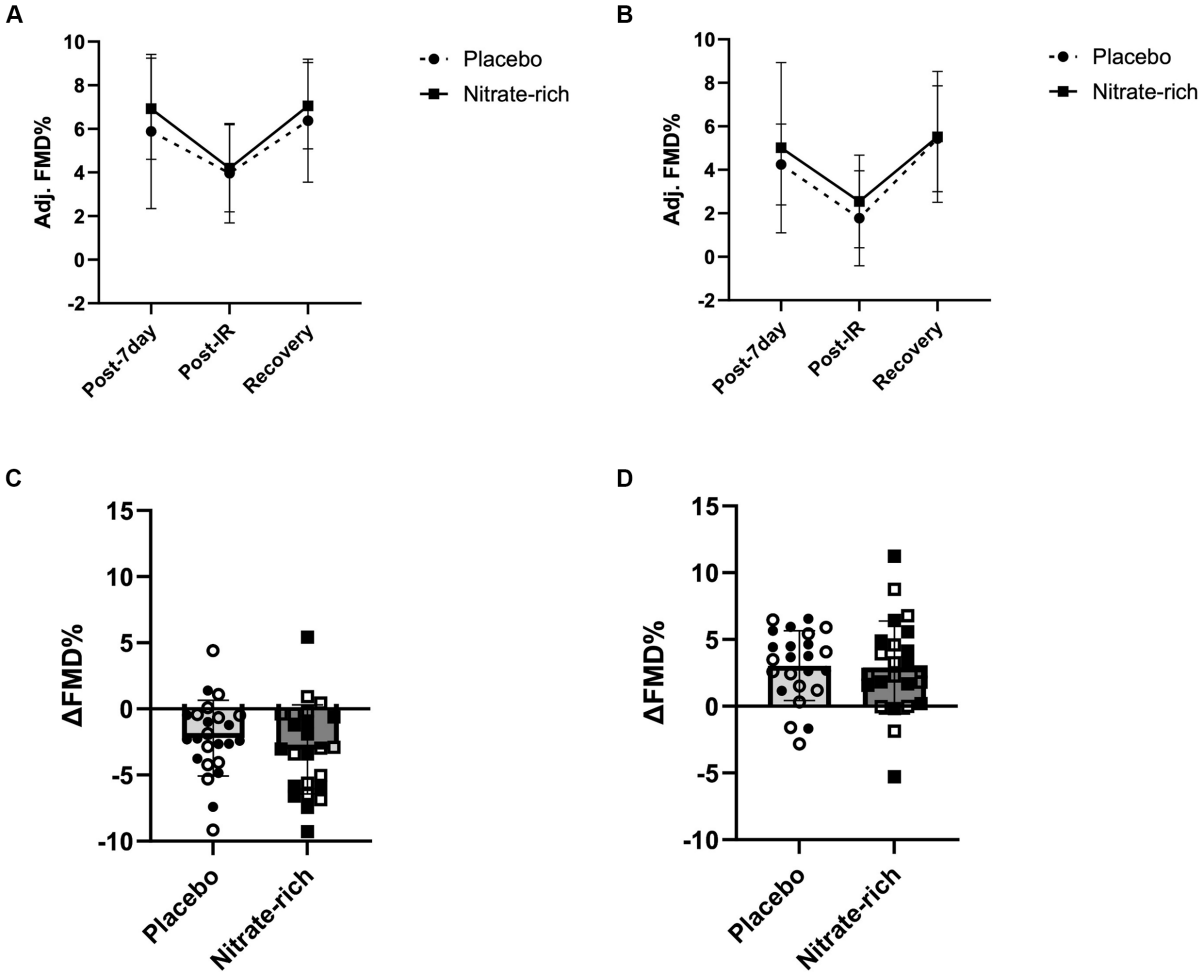
The effects of 7-day nitrate-rich (squares) and nitrate-depleted (circles) BR supplementation on brachial artery flow-mediated dilation measured 24 h following the last BR dose (post-7 day), immediately following the IR injury protocol (post-IR), and 15 min later (Recovery). Results of the linear mixed-effects model are presented as mea*n* ± SD for **(A)** 12 early-postmenopausal and **(B)** 12 late-postmenopausal women. The absolute delta between **(C)** post-7-day and post-IR time points and **(D)** post-IR and recovery time points for early-postmenopausal (open circles and squares) and late- postmenopausal (closed circles and squares) women.

Results of the LMM revealed no significant interaction effects for resting adj. FMD (*p* = 0.89, Figure 4; Table 2), post-IR adj. FMD (*p* = 0.57, Figure 5; Table 2), or recovery adj. FMD (*p* = 0.63, Figure 4; Table 2), suggesting that treatment did not affect resting endothelial function or endothelial resistance to IR injury, in either postmenopausal group, 24 h after the final dose of 7-day BR supplementation. However, our results (Figure 5; Table 2) revealed a significant main effect of the menopausal stage for post-IR adj. FMD (Early_placebo_: 3.96 ± 2.28%; Late_placebo_: 1.76 ± 2.18%, Early_nitrate_: 4.19 ± 2.00%, Late_nitrate_: 2.54 ± 2.13%, *p* = 0.019, Table 2) even after adjusting for 24-h habitual nitrate intake prior to testing and baseline adj. FMD. The same results were true for unadjusted FMD (Table 2).

**Table 2:** The effects of 7-day supplementation with nitrate-rich and nitrate-depleted BR on brachial artery flow mediated dilation measured 24 h following the last BR dose (post-7 day), immediately after IR-injury (post-IR), and 15 min later to assess recovery in 12 early- and 12 late-postmenopausal women.

	**Early-postmenopausal**		**Late-postmenopausal**				
**Variables**	**Placebo**	**Nitrate**	**Placebo**	**Nitrate**			
	**Baseline**	**Baseline**	**Baseline**	**Baseline**			
Baseline diameter (mm)	3.48 ± 0.44	3.47 ± 0.35	3.18 ± 0.36	3.25 ± 0.24			
FMD (%)	5.72 ± 1.83	5.13 ± 3.25	4.81 ± 2.81	3.72 ± 2.16			
Adjusted FMD (%)	5.71 ± 1.75	5.08 ± 3.07	4.77 ± 2.69	3.70 ± 2.07			
Time to peak (s)	41.6 ± 8.1	53.2 ± 11.0	59.5 ± 23.1	48.3 ± 19.0			
Shear rate AUC (10^−3^)	19.2 ± 5.6	19.6 ± 9.9	20.7 ± 9.2	21.5 ± 11.6	**p-values**		
**Variables**	**Post-7 day**	**Post-7 day**	**Post-7 day**	**Post-7 day**	**Treatment**	**Group**	Group*Treatment
Baseline diameter (mm)	3.46 ± 0.40	3.50 ± 0.40	3.20 ± 0.31	3.19 ± 0.34	0.54	0.05	0.77
FMD (%)	5.94 ± 3.70	6.95 ± 2.44	4.25 ± 1.93	5.99 ± 2.61	0.08	0.13	0.63
Adjusted FMD (%)	5.88 ± 3.53	6.92 ± 2.32	4.24 ± 1.86	5.02 ± 3.91	0.31	0.070	0.89
Time to peak (s)	43 ± 9.2	50.3 ± 10.1	46.2 ± 11.7	42.1 ± 10.3	0.87	0.63	0.23
Shear rate AUC (10^−3^)	17.7 ± 2.6	19.7 ± 5.3	18.9 ± 8.8	22.6 ± 14.0	0.55	0.86	0.95
**Variables**	**Post-IR**	**Post-IR**	**Post-IR**	**Post-IR**	**Treatment**	**Group**	Group*Treatment
Baseline diameter (mm)	3.68 ± 0.51	3.54 ± 0.39	3.35 ± 0.32	3.24 ± 0.35	0.2	0.046	0.98
FMD (%)	3.99 ± 2.35	4.21 ± 2.08	1.79 ± 2.22	2.59 ± 2.16	0.29	0.021	0.54
Adjusted FMD (%)	3.96 ± 2.28	4.19 ± 2.00	1.76 ± 2.18	2.54 ± 2.13	0.3	0.019	0.57
Time to peak (s)	42.1 ± 7.6	47.4 ± 19.3	40.7 ± 9.2	41.4 ± 9.3	0.31	0.54	0.81
Shear rate AUC (10^−3^)	15.7 ± 4.6	12.4 ± 2.6	20.8 ± 8.5	14.6 ± 5.4	0.45	0.82	0.57
**Variables**	**Recovery**	**Recovery**	**Recovery**	**Recovery**	**Treatment**	**Group**	Group*Treatment
Baseline diameter (mm)	3.48 ± 0.41	3.35 ± 0.37	3.23 ± 0.35	3.11 ± 0.30	0.004	0.06	0.86
FMD (%)	6.41 ± 2.95	7.08 ± 2.09	5.45 ± 2.56	5.54 ± 3.10	0.53	0.19	0.64
Adjusted FMD (%)	6.37 ± 2.82	7.06 ± 1.98	5.43 ± 2.44	5.51 ± 3.00	0.54	0.19	0.63
Time to peak (s)	45.5 ± 14.4	35.1 ± 9.2	49.7 ± 12.0	55.1 ± 23.7	0.84	0.44	0.66
Shear rate AUC (10^−3^)	16.4 ± 3.8	18.2 ± 6.2	19.2 ± 3.1	17.7 ± 6.2	0.88	0.44	0.94

Results of the LMM, with appropriate adjustments for baseline and post-7-day measures, are presented as mea*n* ± SD.

### 3.7 Effects of BR_placebo_ and BR_nitrate_ on the absolute change in macrovascular endothelial function

Our findings revealed a significant main effect of treatment (*p* = 0.042, Figure 4D) for the absolute difference (delta) in resting FMD pre- and post-7-day supplementation, even after adjusting for 24 h habitual dietary nitrate intake prior to testing and baseline FMD. Figure 4D illustrates that the mean difference in resting FMD was 2.21 ± 5.18% [95% CI (0.082, 4.34), *p* = 0.042] between BR_nitrate_ [2.04 ± 3.66%, CI 95% (0.54, 3.55)] and BR_placebo_ [−0.17 ± 3.66%, CI 95% (−1.68, 1.34)] treatment conditions. No significant main or group*treatment interaction effects were present for the absolute change in FMD between baseline and post-7-day timepoints (Figure 4D), post-7-day and post-IR timepoints (Figure 5C), and post-IR and recovery timepoints (Figure 5D).

### 3.8 Effects of BR_placebo_ and BR_nitrate_ on macrovascular shear patterns

A significant group*treatment interaction effect was present for oscillatory shear rate index (OSI) post-IR (*p* = 0.035, data not shown); however, no significant pairwise comparisons were observed. Additionally, a significant group*treatment interaction was observed for the negative shear rate at the recovery timepoint compared to post-IR (*p* = 0.02, data not shown); however, no significant pairwise comparisons were revealed. No significant interaction or main effects were observed for the positive shear rate. All indirect measures of microvascular function and macrovascular blood flow were non-significant (*p* > 0.05, data not shown).

## 4 Discussion

In the present study, we aimed to investigate whether the vascular protective effects of 7-day supplementation with BR_nitrate_ would be maintained 24 h after the final dose (Day 8) and whether this short-term nutraceutical intervention confers menopausal-stage-dependent effects on endothelial function (1) pre-IR (2) post-IR, and (3) plasma NO metabolite concentrations. First, we demonstrate that 7-day BR_nitrate_ supplementation (400 mg NO_3_^−^ per 70 mL x 7 days) improved the absolute change in resting FMD to a greater extent than BR_placebo_. Second, we show that despite a clinically significant increase in resting FMD prior to the IR protocol, 7-day BR_nitrate_ supplementation did not improve endothelial resistance in either postmenopausal group 24 h after the last dose. Our findings highlight that nitrate-mediated postmenopausal endothelial protection may be dependent on the timing of supplementation in relation to IR injury and chronobiological variations in dietary nitrate metabolism.

### 4.1 The protective effects of dietary nitrate supplementation on endothelial resistance to IR injury

In the current study, we found that nitrate-mediated endothelial protection against IR injury was not maintained 24 h after the last dose of 7-day BR_nitrate_ supplementation in healthy, normotensive women at two distinct stages of postmenopause. Previously, we showed that early-postmenopausal women exhibit an exaggerated decline in FMD, in response to the same IR injury protocol, compared to premenopausal women, despite both groups demonstrating comparable basal FMD values (13). The timing hypothesis emphasizes that pharmaceutical and exercise interventions are less efficacious in reducing CVD risk in estrogen-deficient late-postmenopausal women compared to recently postmenopausal women (18, 39). In contrast to this hypothesis, preliminary data from our laboratory suggest that a single, but higher dose of BR_nitrate_ (600 mg NO_3_^−^ per 140 mL, approx. 100 min absorption period) significantly improved endothelial resistance to IR in both early-postmenopausal (27) and late-postmenopausal stages, with resistance in the late-postmenopausal group, potentially attributed to enhanced resting endothelial function prior to the IR protocol (data not published). To extend our previous acute supplementation work, we investigated whether the vascular protective effects of 7-day supplementation with BR_nitrate_ are maintained 24 h after the final dose and whether short-term BR_nitrate_ confers postmenopausal-stage effects on (1) endothelium-dependent vasodilation pre- and post-IR and (2) plasma NO metabolite concentrations. We hypothesized that menopause-induced endothelial dysfunction would be harder to reverse as time since menopause increases. However, contrary to our hypothesis, our results suggest that, regardless of postmenopausal stage, short-term BR_nitrate_ supplementation is sufficient to induce a clinically meaningful (≥1% increase in FMD which is equivalent to a 13% reduction in CVD risk) (38) improvements in resting FMD, regardless of postmenopausal stage. Furthermore, despite this clinically meaningful increase in resting FMD prior to the IR protocol, the decline in post-IR FMD was similar between treatment conditions (Figure 5; Table 2). Collectively, these findings suggest that 1 week of once-daily BR_nitrate_ consumption did not enhance endothelial protection against IR in either postmenopausal group.

These results are potentially explained by the lack of a significant increase in plasma [nitrite] concentration following 7 days of BR_nitrate_ and may be due to chronobiological variations in dietary nitrate metabolism and/or the transient half-life (20–45 min) of peak plasma [nitrite] (40, 41). Additionally, previous studies have shown a significant reduction in plasma and saliva [nitrate and nitrite] within 48 h following 7 days of a high-nitrate diet. These data support the idea that the vascular benefits of chronic and/or short-term high dietary nitrate intake may largely be due to the continual acute effects on NO conversion. Together, our findings imply that nitrate-mediated endothelial protection against IR insult may depend on the *daily therapeutic dose* (due to chronobiological variations in nitrate metabolism) and the *timing of inorganic dietary nitrate consumption in relation to IR*.

### 4.2 The effects of 7-day dietary nitrate supplementation on endothelial resistance to IR injury

There is a general consensus about the principal pathways and primary mechanisms involved in the conversion of exogenous dietary nitrate to circulating nitrite and NO. In the present study, plasma nitrate significantly increased across timepoints with BR_nitrate_ supplementation in both groups, but not with BR_placebo_. These results imply that the BR_placebo_ treatment contained minimal nitrate (approximately 40 mg nitrate per 70 mL BR_placebo_) and that participants complied with the 7-day supplementation protocol (Figure 3). Due to chronobiological variations in nitrate metabolism, normalizing nitrate load to body mass or adjusting the nitrate load to a pre-set physiologically efficacious plasma threshold for each participant (40) may be necessary to observe significant increases in plasma [nitrite] and NO-mediated vasodilation in this population.

Unexpectedly, we observed a similar increase in plasma nitrite after 7 days of juice consumption in both treatment conditions. Significant variations in [nitrite] attained in the plasma per unit amount of nitrate administered are impacted by gut bacteria capacity (42, 43), high thiocyanate consumption (co-consumption with dietary nitrate can reduce the capacity of nitrate to nitrite conversion) (44), and age- and menopause-induced biological variations in oxidative distress and NO sequestration (43). It is possible that the antioxidant capacity (45, 46) of BR_placebo_ [closely matched to the total polyphenols in BR_nitrate_: 2126.28 ± 113.9 μg FAE/mL (47, 48)] reduced systemic NO sequestration to a similar extent as BR_nitrate_, thus leading to reduced plasma [peroxynitrite] (45, 46, 49) and higher plasma [nitrite] in both treatment conditions. Additionally, both treatments minimally increased plasma [nitrite] (by approximately 50 nM, Figure 3) to the homeostatic plasma nitrite level (110 ± 36 nM NO_2_^−^, 55–210 nM NO_2_^−^) that is commonly observed in healthy adults (46, 50, 51); therefore, BR juice may restore oxidative eustress regardless of [nitrate] and may primarily be driven by betanin antioxidant capacity (52). Moreover, only BR_nitrate_ induced a clinically significant improvement in resting FMD (Figure 4; Table 2). These findings suggest that the synergistic interplay between nitrate and other biologically active phytonutrients in BR is imperative for inducing clinically meaningful enhancements in resting FMD; however, regardless of dietary nitrate concentration, BR remains a promising nutraceutical to increase plasma NO metabolites in postmenopausal women (51).

### 4.3 Endothelial resistance is dependent on the timing of dietary nitrate supplementation in relation to IR insult

The performance-enhancing effects of dietary nitrate are attributed to both nitrite and NO-mediated enhancements in mitochondrial efficiency (53) and/or enhanced muscle blood flow (54). Previous studies have observed a significant increase in plasma [nitrite] relative to the increase in plasma [nitrate] [Δ(nitrite)/Δ(nitrate) ratio] when using similar or lower BR_nitrate_ doses (3.1–4.2 mmol) than employed in the current study (54, 55). However, interestingly, neither postmenopausal group achieved the recommended performance enhancing Δ[nitrite]/Δ[nitrate] ratio (1.0–1.2) following short-term supplementation with BR_nitrate_. Prior evidence from our laboratory and others demonstrates that peak plasma [nitrite] (23, 27) and the performance-enhancing Δ[nitrite]/Δ[nitrate] ratio is achieved 1–3 h post-nitrate consumption (with 4.2–8.4 mmol NO_3_^−^) and returns to baseline concentration approximately 10 h later (40). Additionally, between-day changes in body posture (56), oral temperature, and pH (57) have been reported to influence dietary nitrate metabolism in healthy adults. Therefore, it is possible that 7-day BR_nitrate_ sufficiently increased peak plasma [nitrite] in the 24 h following the last BR dose, prior to the post-supplementation blood draw and vascular assessments. While further mechanistic evidence is warranted, nitrate-mediated improvements in endothelial resistance may be confounded by the timing of supplementation in relation to IR, such that an acute dose 1–3 h before IR may elicit greater endothelial protection in postmenopausal women (27).

### 4.4 The effect of 7-day BR_nitrate_ on macrovascular hemodynamics

Previous findings from our laboratory demonstrate increased peripheral macrovascular retrograde and oscillatory shear in post-menopausal compared to perimenopausal women (27). Findings from the present study demonstrated no significant effects of 7-day BR_nitrate_ supplementation on the oscillatory shear rate. These results could be explained by the similar increase in plasma [nitrite] with both treatment conditions. Furthermore, previous studies have found reductions in retrograde and oscillatory shear following 4 weeks of daily combined inorganic nitrate and nitrite (~4.03 mmol NO_3_^−^ + ~0.29 mmol NO_2_^−^ per 178–237 mL) supplementation in older men and women (58). Therefore, it is possible that a longer supplementation duration and/or addition of dietary nitrite in the supplement is necessary to see clinical improvements in postmenopausal macrovascular shear patterns.

### 4.5 Experimental considerations

One key advantage of this study lies in the utilization of an authentic nitrate-depleted supplement (BR_placebo_) to evaluate the effects of dietary nitrate on endothelial resistance against IR injury (54, 55, 59). However, this method constrains our comprehension of the potential synergistic role between dietary nitrate and other bioactive elements present in the supplement. Additionally, a strength of this study was the use of a comprehensive plant- and animal-based foods dietary nitrate–nitrite database to quantify and control for 24-h dietary nitrate consumption prior to pre- and post-supplementation visits. One limitation in the study design is performing two FMD assessments (15 min apart) before the IR protocol. Nevertheless, any crossover design adopted in this study likely accounted for any potential protective effects of ischemic preconditioning. Although hormone concentrations were not measured in our subject pool, the inclusion of such measurements alongside self-reported menopausal status could have facilitated a more accurate classification of participants into the relevant postmenopausal stages. Additionally, the incorporation of a perimenopausal reference group and/or third treatment arm (ex. nitrate-free water) would have permitted further characterization of menopause-induced endothelial dysfunction and would have enabled comparisons of plasma NO metabolites following 7-day BR supplementation. Furthermore, measuring circulating oxidative stress biomarkers, albeit systemic and not endothelium-specific, would have provided greater mechanistic insight into the antioxidant potential of short-term beetroot juice supplementation.

## 5 Conclusion

We demonstrate that 7-day nitrate-rich beetroot juice supplementation improved endothelial function to a clinically significant level in postmenopausal women with no difference between early and late menopause. This finding is of particular importance given that prior evidence and the *timing hypothesis* suggest that pharmaceutical interventions are less effective at reversing endothelial dysfunction in women beyond 6 years since menopause. However, despite a clinically meaningful increase in basal endothelial function prior to the IR protocol, short-term BR_nitrate_ supplementation did not enhance endothelial resistance against IR injury in either postmenopausal group. Our findings reinforce the notion that the vascular benefits of chronic and/or short-term high dietary nitrate intake may be largely due to the continual acute effects on NO conversion. Therefore, further investigation into the *optimal daily therapeutic dose of dietary nitrate, chronobiological* var*iations in nitrate metabolism,* and the *timing of supplementation in relation to IR insult* is needed to better understand the effects of nitrate-mediated endothelial protection in postmenopausal women.

## Data availability statement

The original contributions presented in the study are included in the article/supplementary material, further inquiries can be directed to the corresponding author.

## Ethics statement

The studies involving humans were approved by the Pennsylvania State University Institutional Review Board. The studies were conducted in accordance with the local legislation and institutional requirements. The participants provided their written informed consent to participate in this study.

## Author contributions

JD: Conceptualization, Data curation, Formal analysis, Funding acquisition, Investigation, Methodology, Project administration, Validation, Visualization, Writing – original draft, Writing – review & editing. JG: Formal analysis, Investigation, Writing – review & editing. LZ: Data curation, Formal analysis, Software, Writing – review & editing. CB: Data curation, Formal analysis, Software, Writing – review & editing. KP: Formal analysis, Supervision, Writing – review & editing. MS: Investigation, Supervision, Writing – review & editing. EA: Data curation, Formal analysis, Investigation, Resources, Writing – review & editing. DK-S: Resources, Supervision, Writing – review & editing. YS: Conceptualization, Methodology, Supervision, Writing – review & editing. DP: Conceptualization, Funding acquisition, Methodology, Resources, Software, Supervision, Writing – review & editing.

## Footnotes

^1^ http://www.randomization.com
